# Extracellular Vesicles at the Interface of Cancer Therapy and Regenerative Medicine: Biology, Bidirectional Roles, and Engineered Therapeutics

**DOI:** 10.3390/medsci14050519

**Published:** 2026-08-26

**Authors:** Jun-Hyeog Jang

**Affiliations:** Department of Biochemistry, Inha University School of Medicine, Incheon 22212, Republic of Korea; juhjang@inha.ac.kr; Tel.: +82-32-860-9863

**Keywords:** extracellular vesicles, exosomes, mesenchymal stem cells, regenerative medicine, tumor microenvironment

## Abstract

Extracellular vesicles (EVs), including exosomes and microvesicles, are nanoscale membrane-enclosed particles that transfer proteins, lipids, mRNAs, and microRNAs between cells. The same communication system is now being developed in two apparently opposing settings: mesenchymal stem/stromal cell (MSC)-derived EVs are explored as cell-free regenerative therapeutics, whereas tumor-derived EVs can promote metastasis, immune evasion, and treatment resistance while also serving as liquid-biopsy biomarkers. Rather than treating these studies separately, this review compares them along shared mechanistic axes: producer-cell identity and state, luminal cargo, membrane and surface composition (including the biomolecular corona), dose and administration route, biodistribution, and recipient-cell context. This cross-field perspective shows that angiogenesis, immunomodulation, matrix remodeling, and tissue tropism are not intrinsically regenerative or oncogenic; their consequences depend on where, how, and to which cells EV signals are delivered. We review EV biology and MISEV2023-aligned nomenclature, examine bidirectional regenerative and cancer-associated functions, and survey engineering strategies for cargo loading and surface modification together with plant-derived exosome-like nanovesicles. We further compare EVs with lipid nanoparticles, adeno-associated viruses, polymers, and virus-like particles, and discuss how engineering can improve potency and targeting while increasing manufacturing and characterization complexity. Finally, we address tumor-related safety considerations for regenerative EVs and the unresolved biological, technical, manufacturing, and regulatory questions that must be addressed for clinical translation.

## 1. Introduction

Cells exchange information continuously, and one route for this exchange is the extracellular vesicle (EV). EVs are nanoscale, lipid-bilayer–enclosed particles, principally exosomes and microvesicles, released by virtually all cell types and recovered from every biological fluid examined to date, including plasma, urine, saliva, breast milk, cerebrospinal fluid, and synovial fluid [1,2]. First described decades ago as antigen-presenting vesicles secreted by immune cells, EVs were long dismissed as cellular debris or as a disposal route for obsolete membrane proteins [3]. They are now recognized as selective carriers of bioactive cargo that alter the phenotype of recipient cells, and EV research has become an active area of molecular and cellular medicine [1,4].

Vesicle release was first characterized in maturing reticulocytes, where it appeared to serve mainly to discard the transferrin receptor; the demonstration that B lymphocytes secrete vesicles bearing functional peptide–major histocompatibility complex (MHC) class II complexes capable of stimulating T cells provided the first evidence of a signaling function [3]. The subsequent discovery that exosomes carry mRNA and microRNA (miRNA) that remain functional after transfer to recipient cells established EVs as a general mechanism of horizontal molecular exchange [5]. Within two decades, EV research has expanded to encompass developmental biology, neurodegeneration, immunology, infectious disease, cardiovascular medicine, oncology, and regenerative therapy, supported by an increasingly detailed understanding of the molecular machinery that governs vesicle biogenesis, cargo sorting, and secretion [6].

A central feature of EV biology is its duality. The same vesicular communication system is harnessed therapeutically to regenerate tissue and is hijacked pathologically to propagate cancer. MSC-derived EVs have emerged as a leading cell-free regenerative platform, whereas tumor-derived EVs are active participants in metastasis, immune evasion, and treatment resistance and can also be exploited as biomarkers or drug-delivery vehicles. These applications share several potentially useful features, but none should be treated as universal properties of EVs. The lipid bilayer can protect labile cargo, and membrane-associated molecules can influence cell recognition and uptake; however, immunogenicity, circulation time, barrier traversal, and tissue tropism vary with producer-cell source, purification method, membrane and protein-corona composition, dose, administration route, and recipient state. In particular, intravenously administered EVs are commonly cleared rapidly by the mononuclear phagocyte system and accumulate in liver, spleen, and lung, so selective delivery to other tissues generally requires route selection or engineering [7,8,9,10]. EVs therefore lie at the interface of cancer therapy and regenerative medicine, but their value is best understood through these context-dependent determinants rather than through a single set of intrinsic advantages.

A persistent obstacle to consolidating these lessons has been definitional heterogeneity. Because no single marker unambiguously assigns an EV to a specific biogenesis route, and because conventional preparations co-isolate lipoproteins, protein aggregates, and non-vesicular nanoparticles, the term “exosome” has been applied inconsistently across thousands of publications. Careful biochemical dissection has shown that several molecules long regarded as canonical exosome constituents, including certain cytosolic RNA-binding proteins and much of the extracellular DNA in conditioned medium, are in fact secreted through non-vesicular routes [11], and that a distinct class of non-membranous particles termed exomeres coexists with small EVs in the same size range [12]. In response, the International Society for Extracellular Vesicles (ISEV) has issued successive consensus frameworks, most recently the Minimal Information for Studies of Extracellular Vesicles (MISEV2023), to standardize nomenclature, isolation, and characterization and to improve rigor and reproducibility [13,14], complemented by community reporting platforms such as EV-TRACK that make experimental provenance transparent and auditable [15].

In this review, we (i) summarize EV biology, cargo sorting, uptake, and analysis in light of current consensus guidance; (ii) compare regenerative and oncogenic EV biology directly using shared determinants of activity; (iii) examine how the same effector programs—angiogenesis, immune modulation, cell survival, and matrix remodeling—can produce beneficial repair or tumor-supportive outcomes; and (iv) survey engineering, botanical sourcing, manufacturing, and regulatory issues relevant to both fields. The distinctive objective is not simply to place two mature studies side by side. Instead, we use their intersection to extract cross-field lessons: oncology highlights safety liabilities of pro-reparative signaling and provides stringent targeting and liquid-biopsy analytical frameworks, whereas regenerative medicine provides potency, local-delivery, biomaterial, and manufacturing concepts that can inform cancer EV therapeutics. This comparative framework also identifies where EV engineering may add value over synthetic or viral delivery systems and where it introduces additional product complexity.

## 2. Biology, Classification, and Analysis of Extracellular Vesicles

### 2.1. Biogenesis and Subtypes

**Figure 1:**
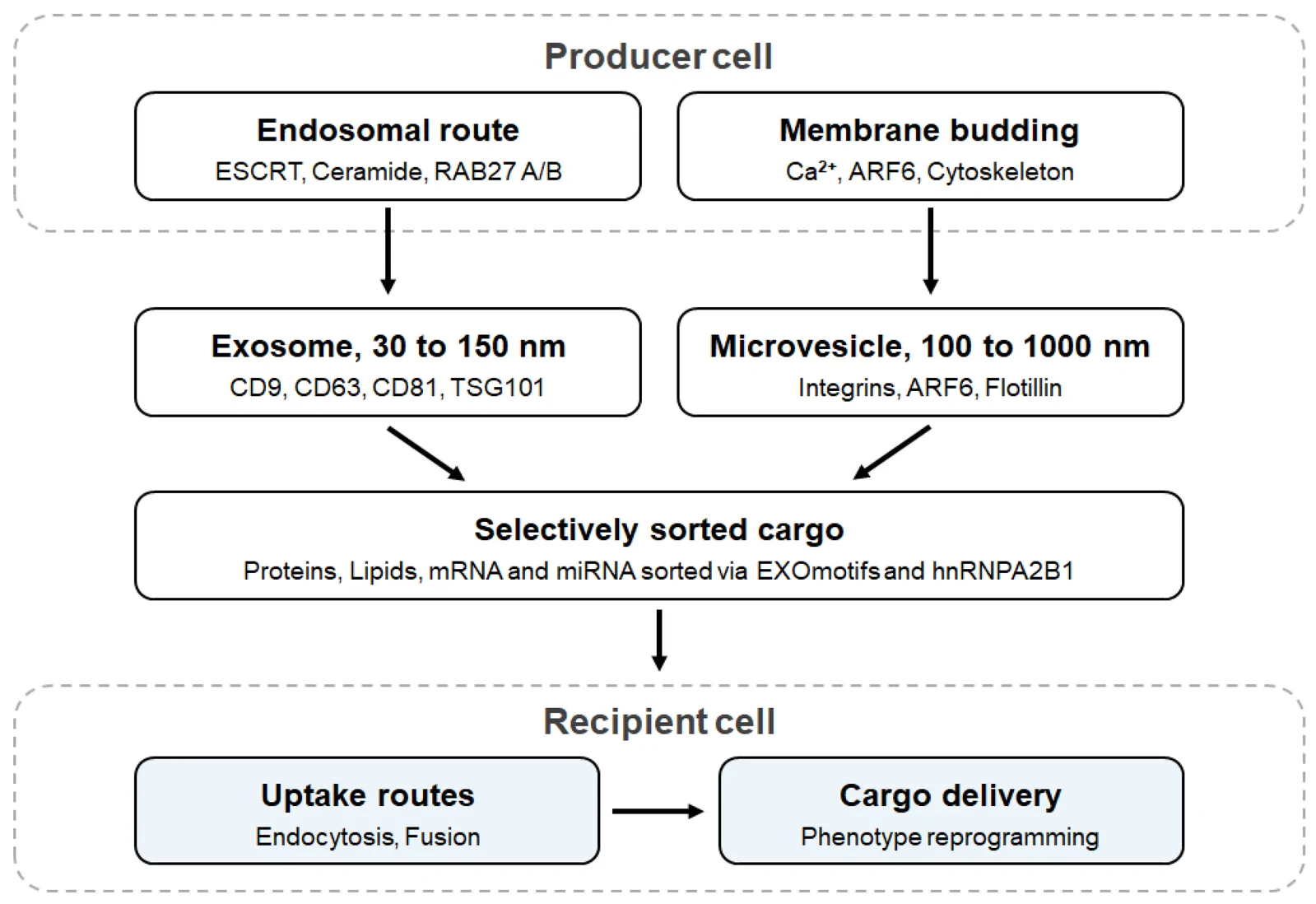
Biogenesis, cargo, and uptake of extracellular vesicles. Exosomes originate in the endosomal system, where intraluminal vesicles form within multivesicular bodies and are released on fusion with the plasma membrane, whereas microvesicles bud directly outward from the plasma membrane. Both classes carry selectively sorted protein, lipid, and RNA cargo that is delivered to recipient cells after uptake, most commonly by endocytosis or membrane fusion.

EVs are a heterogeneous family of phospholipid-bilayer particles secreted by essentially all prokaryotic and eukaryotic cells [2,16]. Two principal biogenesis routes are recognized (Figure 1). Exosomes (typically ~30–150 nm) originate in the endosomal system: inward budding of the limiting membrane of multivesicular bodies generates intraluminal vesicles that are released when the multivesicular body fuses with the plasma membrane, a process orchestrated by the endosomal sorting complex required for transport (ESCRT) and by ESCRT-independent machinery [2,17]. Microvesicles (ectosomes; ~100–1000 nm) instead bud directly outward from the plasma membrane, a process driven by calcium-dependent loss of phospholipid asymmetry, cytoskeletal remodeling, and small GTPase activity, whereas apoptotic bodies are shed by dying cells (Table 1).

The molecular detail underlying these routes has clarified how vesicle production is regulated and how it can be manipulated experimentally. The ESCRT-0 through ESCRT-III complexes, together with accessory factors such as ALIX and VPS4, recognize ubiquitinated cargo and execute membrane scission; syntenin–ALIX–syndecan signaling provides a parallel, tetraspanin-associated route [6]. ESCRT-independent budding can be driven by the sphingolipid ceramide, generated by neutral sphingomyelinase, which imposes negative membrane curvature sufficient to bud intraluminal vesicles even when ESCRT function is impaired [18]. Trafficking of multivesicular bodies to the plasma membrane and their fusion with it require Rab GTPases and SNARE proteins; RAB27A and RAB27B act at distinct steps, with RAB27A controlling docking of multivesicular bodies at the plasma membrane and RAB27B governing their transfer from the Golgi region, and silencing either markedly reduces exosome output [19]. These molecules are the main targets for experimentally increasing or suppressing EV release, and they are used in both manufacturing and mechanistic studies.

Because markers that unambiguously assign subcellular origin remain elusive, MISEV2023 recommends defining EVs operationally by physical attributes (e.g., small versus large EVs) and reporting their provenance and characterization in full [13,20]. This recommendation is reinforced by evidence that preparations of “small EVs” are themselves compositionally heterogeneous, comprising at least two exosome subpopulations of distinct size and cargo alongside non-vesicular exomeres enriched in metabolic enzymes and glycan-processing proteins [12]. Investigators should therefore treat subtype assignments as provisional unless supported by orthogonal separation and single-particle analysis.

### 2.2. Cargo Composition and Selective Sorting

EVs mediate horizontal information transfer between cells. Their luminal and membrane cargo, which includes signaling proteins, enzymes, lipids, mRNAs, and regulatory miRNAs, can be delivered to recipient cells and remain functional: an early study showed that exosomal mRNAs and miRNAs are transferred between cells and translated or act as gene regulators in the recipient [5]. Tumor cells similarly export RNA and protein cargo that promote growth and angiogenesis and that betray the molecular state of the originating cell [21]. This capacity for protected and selective molecular delivery underlies both the physiological and pathological roles of EVs and explains their interest as a therapeutic modality [1].

Cargo incorporation is selective. The EV proteome is enriched for tetraspanins (CD9, CD63, CD81), ESCRT components (TSG101, ALIX), heat-shock proteins, integrins, and MHC molecules, while being depleted of endoplasmic reticulum, Golgi, and nuclear proteins, an asymmetry consistent with regulated sorting [6]. The EV lipidome is likewise distinctive, enriched in cholesterol, sphingomyelin, ceramide, and phosphatidylserine relative to the parental plasma membrane, which contributes to vesicle rigidity, stability in biological fluids, and recognition by phosphatidylserine receptors on recipient cells. RNA sorting is similarly directed: specific sequence motifs (“EXOmotifs”) in miRNAs are recognized by the RNA-binding protein hnRNPA2B1, whose sumoylation controls miRNA loading into exosomes, providing one molecular explanation for why exosomal miRNA profiles differ systematically from those of the producing cell [22]. The same motifs and adaptor proteins can be used to load therapeutic RNAs into engineered vesicles.

Two caveats apply. First, rigorous fractionation shows that a substantial fraction of the nucleic acid and protein recovered in crude EV pellets is not vesicle-associated at all, including most extracellular DNA, histones, and several abundant RNA-binding proteins [11]; functional attributions based on unfractionated preparations should therefore be interpreted cautiously. Second, EV composition is highly plastic: producer-cell type, passage number, confluence, oxygen tension, culture medium, and inflammatory or other stimulation states can alter luminal RNA/protein cargo as well as membrane lipid and protein composition [22,23,24,25,26,27]. After release, adsorption of a biomolecular corona can further modify the effective EV surface and influence cellular uptake [10]. This plasticity is an opportunity for potency enhancement but also a major source of batch-to-batch and context-dependent variability.

### 2.3. Uptake, Biodistribution, and Functional Delivery

An EV must reach and productively deliver cargo to a recipient cell in order to act. Uptake proceeds through multiple, partially redundant routes: clathrin-mediated endocytosis, caveolin-dependent endocytosis, lipid-raft-associated internalization, macropinocytosis, phagocytosis, and direct fusion with the plasma membrane, with the dominant pathway determined by the molecular composition of both the vesicle and the recipient cell [28]. Integrins, tetraspanins, proteoglycans, lectins, and adsorbed corona proteins can contribute to docking and uptake, while recipient-cell receptor density, activation state, and local microenvironment further shape these interactions [10,28]. A practical consequence is that most internalized EVs are routed to the endolysosomal compartment, where cargo may be degraded before reaching the cytosol; endosomal escape and productive cytosolic delivery are therefore important rate-limiting steps, particularly for nucleic-acid therapeutics.

Whole-animal imaging has defined the pharmacokinetic behavior of systemically administered EVs. After intravenous injection, many EV preparations are cleared rapidly from plasma and distribute predominantly to liver, spleen, lung, and other mononuclear-phagocyte-system-rich organs; quantitative patterns vary with producer-cell source, purification method, route, dose, labeling strategy, and surface engineering [7,9]. Thus, reports of long circulation, broad barrier crossing, or intrinsic tissue selectivity should not be generalized across EV products. Local or loco-regional administration (for example, topical, intra-articular, intramyocardial, or intranasal delivery) can reduce systemic sequestration for some indications, whereas systemic delivery to non-clearance-organ targets often requires deliberate engineering and quantitative biodistribution testing. Dosing should be reported using defined particle, protein, or lipid metrics together with a mechanism-linked potency assay rather than arbitrary units of conditioned medium [7,9,23].

### 2.4. Isolation, Characterization, and Standardization

A practical limitation is the difficulty of isolating and characterizing EVs reproducibly. Differential ultracentrifugation, density-gradient separation, size-exclusion chromatography, precipitation, and immunoaffinity capture each trade purity against yield, and no single method is ideal for all applications (Table 2) [29,30]. Differential ultracentrifugation remains the most widely used reference method but co-sediments protein aggregates and lipoproteins and can damage vesicles through mechanical stress; density-gradient flotation improves purity at substantial cost in yield and throughput; size-exclusion chromatography preserves vesicle integrity and biological activity but produces dilute eluates requiring concentration; polymer precipitation is fast and scalable but yields impure preparations; and immunoaffinity capture delivers subtype-specific material at the price of capture bias, low yield, and high reagent cost.

Characterization requires similar care. Current consensus requires orthogonal evidence at three levels: quantification of particle number and size distribution (nanoparticle tracking analysis, tunable resistive pulse sensing, or single-particle interferometric imaging), demonstration of vesicular morphology by electron or atomic force microscopy, and immunodetection of at least one transmembrane marker and one cytosolic marker, together with a negative marker to exclude co-isolated contaminants [13,14]. Bulk assays inevitably average across heterogeneous populations, and single-EV methods, including imaging flow cytometry, high-resolution nanoflow cytometry, and asymmetric flow field-flow fractionation, are increasingly required to resolve genuinely distinct subsets and to distinguish vesicles from non-vesicular nanoparticles [12].

Microfluidic platforms have recently emerged to address the throughput and reproducibility limitations of conventional workflows, enabling rapid, low-volume, high-purity capture and on-chip analysis with reduced batch-to-batch variability, which is particularly important for clinical-grade production and for point-of-care diagnostics [31]. Layered onto these methods, the MISEV2018 and MISEV2023 guidelines provide community standards for nomenclature, minimal experimental requirements, and transparent reporting, without which cross-study comparison and regulatory evaluation are untenable [13,14]. Independent analysis of the published literature through the EV-TRACK knowledgebase has documented how frequently essential experimental parameters go unreported and has provided a practical mechanism, the EV-METRIC completeness score, for authors, reviewers, and readers to assess methodological transparency [15]. Together, these tools support the development of EVs as a defined biomedical product class [32].

## 3. Extracellular Vesicles in Regenerative Medicine

### 3.1. MSC-Derived EVs as a Cell-Free Therapeutic Platform

Most regenerative EV research has used MSCs. It has long been recognized that the benefits of MSC transplantation correlate poorly with donor-cell engraftment and persistence, implicating paracrine mediators rather than cellular replacement as a major mechanism of action. MSC-derived EVs can recapitulate many of these paracrine, trophic effects. As a cell-free product they avoid several risks intrinsic to administration of viable proliferative cells, including uncontrolled proliferation, ectopic differentiation, and pulmonary entrapment, and they can offer improved storage and dose definition and permit terminal sterile filtration [23,33]. However, low immunogenicity, favorable circulation, and tissue selectivity should be demonstrated for each product rather than assumed, because these properties depend on producer source, processing, route, dose, and recipient context. MSC-EVs are therefore attractive regenerative candidates, but their advantage over cells or synthetic carriers is indication- and product-specific.

MSC-EVs can be obtained from bone marrow, adipose tissue, umbilical cord and Wharton’s jelly, placenta, and dental pulp, and these sources differ in vesicle yield per cell, in miRNA and protein composition, and in potency across disease models, with umbilical-cord and adipose sources generally offering higher expansion capacity and bone-marrow sources the longest clinical track record. Recognizing that this diversity, combined with heterogeneous production methods, has produced a fragmented and sometimes contradictory literature, an ISEV working group has issued specific guidance for defining MSC-derived small EVs intended for therapeutic use, emphasizing rigorous characterization of both the producer cells and the vesicle product, and the development of quantitative potency assays tied to the intended mechanism of action [23]. Adherence to that framework is important for translational work.

### 3.2. Molecular Mechanisms of EV-Mediated Repair

MSC-EVs accelerate repair by engaging several core programs of healing simultaneously [24,25,26,33,34,35,36]. Their immunomodulatory activity can shift macrophage phenotypes toward reparative states, suppress effector T-cell responses, support regulatory T-cell activity, and reduce pro-inflammatory cytokine signaling. Their pro-angiogenic effects can involve vascular endothelial growth factor, angiopoietins, and pro-angiogenic miRNAs that stimulate endothelial proliferation, migration, and tube formation. Pro-survival and proliferative effects converge on pathways including PI3K/AKT, ERK, and STAT3 in resident stromal and parenchymal cells, while transfer of Wnt ligands can activate beta-catenin signaling required for keratinocyte and fibroblast responses in cutaneous repair [34]. The relative contribution of each pathway varies with MSC source, priming condition, EV preparation, dose, and target tissue, reinforcing the need for mechanism-linked potency assays rather than a single universal MSC-EV mechanism.

An early example of mechanistic dissection comes from the cardiac literature. EVs secreted by human embryonic stem cell-derived MSCs reduced infarct size in a mouse model of myocardial ischemia–reperfusion injury, establishing that a cell-free fraction of MSC conditioned medium was sufficient for cardioprotection and directing attention to the specific proteins and RNAs responsible [37]. Comparable causal experiments in skin identified Wnt4 delivered by umbilical-cord MSC exosomes as a necessary mediator of re-epithelialization, since depleting Wnt4 abolished the therapeutic effect [34]. Studies that demonstrate both the sufficiency and the necessity of a defined EV constituent provide the basis for potency assays and regulatory documentation.

### 3.3. Tissue-Specific Applications

In cutaneous wound models, MSC-EVs attenuate inflammation, promote angiogenesis, stimulate fibroblast and keratinocyte proliferation and migration, and enhance extracellular-matrix production and remodeling, thereby shortening healing time and improving the quality of the regenerated tissue, including reduced scar formation and more organized collagen deposition [35]. These effects are most pronounced in models of impaired healing (diabetic, ischemic, and irradiated wounds), where endogenous repair signaling is deficient.

In joints, MSC-EVs promote chondrocyte proliferation, inhibit chondrocyte apoptosis, dampen pro-inflammatory cytokines and catabolic matrix metalloproteinases, and support cartilage-matrix redeposition, attenuating the progression of osteoarthritis in preclinical models [36]. Analogous pro-angiogenic, immunomodulatory, and pro-osteogenic effects, including stimulation of osteoblast differentiation and coupling of angiogenesis to osteogenesis, support bone regeneration and the healing of critical-size defects [33]. Beyond the musculoskeletal system, MSC-EVs have shown activity in preclinical models of myocardial infarction [37], acute kidney injury, acute lung injury and pulmonary fibrosis, hepatic injury, stroke, spinal cord injury, and peripheral nerve regeneration.

### 3.4. Potency Enhancement: Preconditioning and Biomaterial-Assisted Delivery

Because EV cargo mirrors the physiological state of the producer cell, culture conditions can be used deliberately to enhance potency. Hypoxic preconditioning enriches MSC-EVs in hypoxia-inducible factor-dependent pro-angiogenic mediators [24]; priming with interferon-γ, tumor necrosis factor-α, or lipopolysaccharide amplifies immunomodulatory activity [25,26]; three-dimensional culture in spinner flasks, hollow-fiber bioreactors, or on microcarriers increases both vesicle yield per cell and, in several reports, bioactivity relative to two-dimensional culture [27]. These strategies enhance potency without genetic modification, which simplifies the regulatory path relative to engineered products, although they also introduce additional process parameters that must be controlled and reported.

A second practical constraint is residence time. Free EVs applied to a wound bed or injected into a joint are cleared within hours to a few days, whereas tissue repair unfolds over weeks [38]. Incorporating EVs into hydrogels, decellularized matrices, electrospun scaffolds, or bioprinted constructs provides sustained, localized release, protects vesicles from degradation, and in several comparative studies substantially improves outcomes relative to bolus delivery at the same total dose [38,39]. Scaffold-based delivery is also compatible with existing surgical practice in tissue engineering.

### 3.5. Clinical Translation in Regeneration

Early clinical experience has been encouraging with respect to safety. A widely cited compassionate-use case documented rapid and durable improvement in a patient with steroid-refractory acute graft-versus-host disease treated with escalating doses of MSC-derived exosomes, with no infusion-related toxicity [40]; MSC-EV preparations were subsequently administered intravenously in an open-label cohort of patients with severe COVID-19, again without serious adverse events attributable to the product [41]. In regenerative indications specifically, topical application of umbilical-cord MSC EVs to chronic wounds and intra-articular delivery of bone-marrow MSC EV products in osteoarthritis have been reported with favorable safety profiles and preliminary signals of efficacy [33,36].

Progress is nonetheless constrained by heterogeneity in EV source, isolation method, dose definition, administration route, and outcome reporting, and most evidence still derives from animal models and small or uncontrolled human studies [33,35,36]. The ISEV position paper on clinical application of EV-based therapeutics set out core requirements: defined and traceable starting material, reproducible and scalable manufacturing under good manufacturing practice (GMP), quantitative release criteria, and a potency assay linked to the proposed mechanism [42]. Those requirements remain central gaps between the current evidence base and licensure. Standardized, adequately powered randomized studies with pre-specified endpoints, product-specific biodistribution and safety assessment, and longitudinal follow-up are required [35,36,42].

## 4. Extracellular Vesicles in Cancer

### 4.1. Tumor-Derived EVs, the Pre-Metastatic Niche, and Organotropism

In oncology, EVs contribute to tumor progression. Tumor-derived EVs remodel both local and distant microenvironments: they activate stromal fibroblasts into cancer-associated fibroblasts, stimulate angiogenesis, increase vascular permeability, induce epithelial–mesenchymal transition in neighboring epithelium, transfer oncogenic proteins and nucleic acids between tumor subclones, and recruit bone-marrow–derived cells to establish immunosuppressive pre-metastatic niches that prime future sites of metastasis [43,44,45]. Because this signaling is continuous and systemic, it extends the influence of a primary tumor beyond its anatomical boundaries.

Several studies have established causality. Melanoma exosomes were shown to educate bone-marrow progenitors toward a pro-metastatic phenotype through transfer of the receptor tyrosine kinase MET, increasing metastatic burden, whereas depletion of RAB27A to suppress exosome release reduced it [46]; pancreatic-cancer exosomes carrying macrophage migration inhibitory factor were shown to initiate hepatic pre-metastatic niche formation by inducing transforming growth factor-β release from Kupffer cells and fibronectin deposition by hepatic stellate cells [47]. The integrin repertoire displayed on tumor EVs also influences organotropic metastasis: exosomal integrins α6β4 and α6β1 associate with lung tropism and αvβ5 with liver tropism, and redirecting integrin expression redirects the site of metastasis, providing a molecular explanation for long-observed patterns of organ-specific spread [48]. Together these findings indicate that EVs are causal effectors of metastasis and therefore potential therapeutic targets.

### 4.2. Immune Evasion

Tumor EVs suppress anti-tumor immunity through several complementary mechanisms [45,49,50]. Tumor EVs can carry FasL and TRAIL that promote apoptosis of activated T cells, NKG2D ligands that downregulate activating receptors on natural killer cells, transforming growth factor-beta that favors regulatory T-cell programs, and the ectonucleotidases CD39 and CD73 that generate immunosuppressive adenosine in the tumor microenvironment. They can also expand or activate myeloid-derived suppressor cells and skew monocyte differentiation away from effective antigen presentation. The magnitude of these effects depends on tumor type, EV subpopulation, local concentration, biodistribution, and the state of recipient immune cells, emphasizing that immune suppression is a property of defined tumor-EV contexts rather than of EVs as a class.

The best-characterized example is exosomal PD-L1. Tumor cells export exosomes that display the immune-checkpoint ligand PD-L1 on their surface in the correct topological orientation; circulating exosomal PD-L1 engages PD-1 on T cells systemically, including in lymph nodes before cytolytic T cells ever contact the tumor, and its abundance in plasma, together with its dynamic change during therapy, tracks with response to anti-PD-1 treatment in melanoma [49]. Genetic suppression of exosomal PD-L1 in prostate cancer and colorectal cancer models induces systemic anti-tumor immunity and durable immunological memory, sensitizes otherwise unresponsive tumors, and acts additively with antibody checkpoint blockade, identifying exosomal PD-L1 as a resistance mechanism, a predictive biomarker, and a therapeutic target [50].

### 4.3. Drug Resistance

Tumor EVs also transfer determinants of therapeutic failure. They export cytotoxic drugs directly out of the cell, as shown for cisplatin sequestration and exosomal efflux in resistant ovarian carcinoma; they transfer drug-efflux transporters such as P-glycoprotein, anti-apoptotic proteins, and regulatory RNAs from resistant to sensitive subpopulations, propagating a resistant phenotype horizontally through a genetically heterogeneous tumor; and they modify the stroma so that it supports survival under treatment [43,44].

EVs additionally act as decoys for antibody therapeutics. Exosomes released by HER2-overexpressing breast-cancer cells display full-length HER2, bind trastuzumab, and reduce the antibody available to engage tumor cells, blunting its anti-proliferative activity in vitro in proportion to exosome concentration, an effect amplified in the presence of serum from patients with advanced disease [51]. Analogous sequestration has been described for other therapeutic antibodies whose targets are exosome-associated. Because these mechanisms operate in parallel with classical genetic resistance, tumor EVs are themselves a potential therapeutic target, and strategies have been proposed to inhibit EV biogenesis, block EV uptake, or remove circulating tumor EVs.

### 4.4. EVs as Liquid-Biopsy Biomarkers

EVs are also useful as diagnostic analytes. Because EVs are abundant and stable in body fluids, are protected from ribonuclease and protease degradation, and carry source-cell molecular signatures, they are well suited to minimally invasive “liquid biopsy.” They are present in far greater numbers than circulating tumor cells, they carry intact protein and RNA in addition to DNA; and unlike circulating tumor DNA, which is released predominantly from dying cells, they are actively secreted by living tumor cells, so they report on the viable tumor compartment [45,52].

Several specific analytes support this approach. Glypican-1–positive circulating exosomes distinguished patients with pancreatic ductal adenocarcinoma from healthy individuals and those with benign pancreatic disease with high specificity, and detected early lesions in a preclinical model [53]. Systematic proteomic profiling of EVs and particles from a large panel of human tumors and biofluids subsequently identified pan-cancer and tumor-type–specific protein signatures capable of classifying cancer type and tissue of origin, indicating that EV-based classification is feasible across multiple malignancies [54]. Clinical implementation is already underway in urology: a three-gene urinary exosomal RNA assay (ERG, PCA3, and SPDEF) improved discrimination of high-grade from low-grade prostate cancer over standard clinical variables at initial biopsy and has been prospectively evaluated as a tool to avoid unnecessary biopsies [55].

Beyond detection, EV analytes support molecular subtyping, longitudinal monitoring of treatment response, and detection of resistance mutations, including the EGFRvIII transcript in glioblastoma-derived vesicles recoverable from serum [21]. The principal obstacles to broader adoption are pre-analytical: blood collection tube type, time to processing, centrifugation protocol, and storage temperature all measurably alter EV yield and composition, and standardization of these variables is as important to clinical validity as the analytical assay itself.

## 5. Bidirectional Biology: Shared Programs, Divergent Outcomes, and Cross-Field Lessons

**Figure 2:**
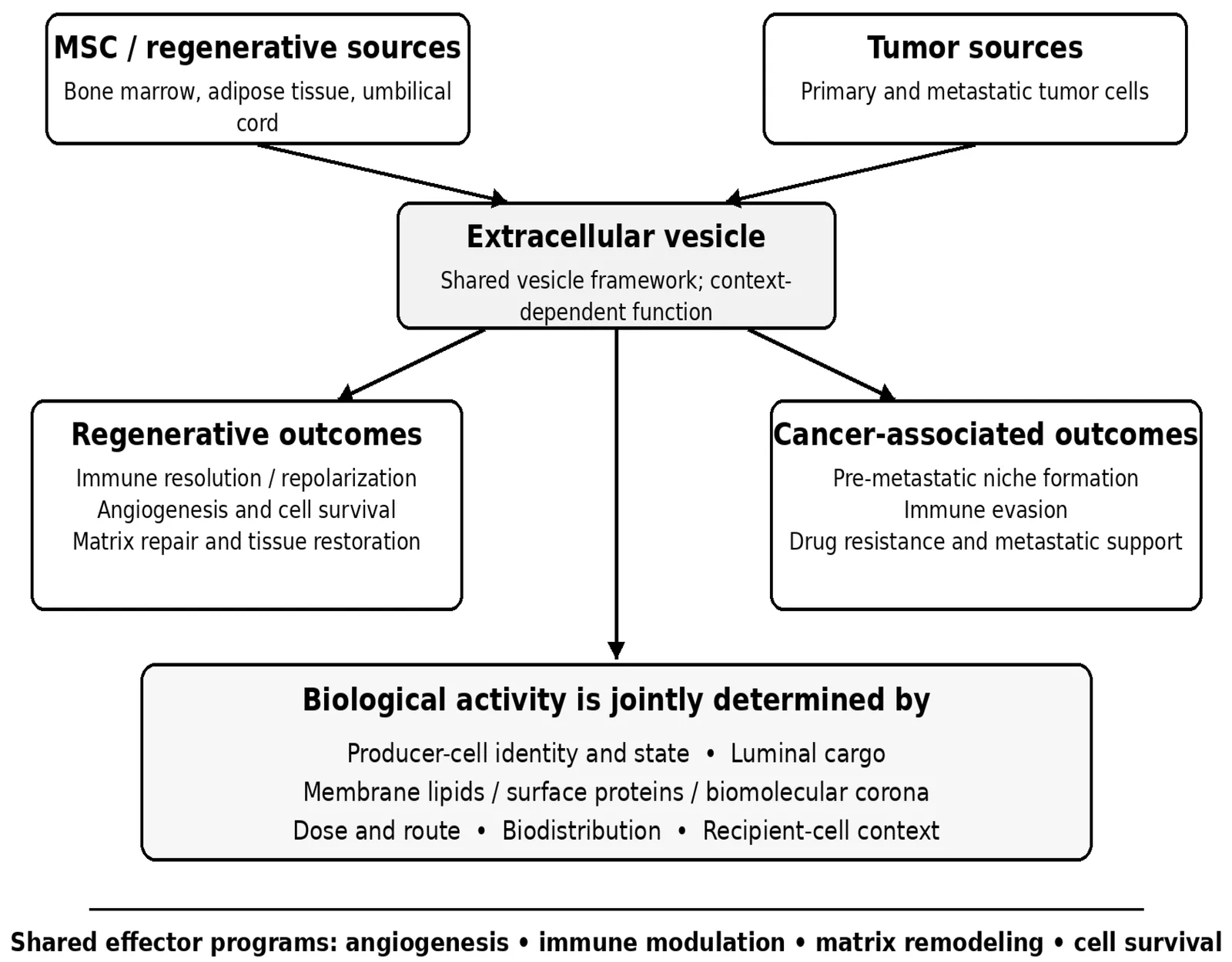
Bidirectional roles of extracellular vesicles and the determinants of biological outcome. EVs from regenerative and tumor sources use overlapping effector programs, but their effects are not determined by source or cargo alone. Producer-cell state, luminal cargo, membrane lipids and surface proteins, the biomolecular corona, dose and route, biodistribution, and recipient-cell context jointly determine whether shared programs such as angiogenesis, immune modulation, matrix remodeling, and cell survival support tissue repair or tumor progression.

EV biological activity is not determined by source and cargo alone. It emerges from the interaction of producer-cell identity and physiological state, luminal cargo, membrane lipid and surface-protein composition, the biomolecular corona acquired after release, administered dose and route, biodistribution, and recipient-cell phenotype (Figure 2) [7,9,10,45]. These determinants explain why the same core programs can produce opposite outcomes: angiogenesis can revascularize an ischemic wound yet support tumor growth; immune suppression can resolve damaging inflammation yet shield malignant cells; matrix remodeling can restore tissue architecture yet facilitate invasion; and pro-survival signaling can preserve injured parenchyma yet promote treatment resistance.

Putting cancer and regenerative EV biology into the same framework therefore changes how each field should interpret efficacy. Oncology shows that pro-reparative programs are potential liabilities when delivered to a permissive tumor microenvironment, while regenerative studies show that route, local retention, biomaterial-assisted delivery, and transient exposure can be used to harness the same pathways spatially and temporally. Methodological lessons also move in both directions: tumor-EV research has developed stringent approaches to molecular subtyping, organotropism, and longitudinal liquid-biopsy analysis, whereas regenerative EV research has emphasized donor-cell qualification, mechanism-linked potency assays, and sustained local delivery. These cross-field lessons support rational engineering, but they also argue against assuming universal EV properties such as intrinsic targeting, long circulation, barrier penetration, or low immunogenicity; each should be demonstrated for the specific product and indication [7,8,9,23,56].

The duality also creates a practical safety question for regenerative medicine: could an EV product designed to stimulate angiogenesis, immune resolution, or cell survival inadvertently support an occult or residual malignancy? Available preclinical evidence is mixed, with MSC-EVs reported to suppress tumor growth in some models and support it in others; a systematic review found substantial heterogeneity related to MSC tissue source, EV preparation or modification, dose and schedule, tumor model, and study design [57]. Donor tissue and culture/priming conditions can change cargo and surface composition, repeated or high systemic doses can alter tissue exposure, and the recipient context—including active or recent cancer, premalignant disease, immune status, and tumor-specific susceptibility—can shift the balance between repair and tumor support [23,33,45,57]. Consequently, risk cannot be inferred from the label ‘MSC-EV’ alone.

For regenerative trials, a risk-adapted strategy should therefore include source- and batch-specific profiling of pro-angiogenic, immunosuppressive, and oncogenic signaling activities; mechanism-linked potency assays accompanied, where biologically relevant, by tumor-support or transformation-safety assays; explicit justification of dose, schedule, and route; and prospective handling of patients with active or recent malignancy through exclusion or stratification according to indication and evidence. Long-term surveillance for incident or recurrent malignancy is particularly important for products intended for repeated administration. This safety framework is a direct lesson from combining oncology and regeneration: the same biological programs that constitute therapeutic potency in injured tissue may constitute hazard in a tumor-permissive context [45,57].

## 6. Engineered and Plant-Derived EVs as Next-Generation Therapeutics

### 6.1. Cargo Loading and Surface Engineering

Natural EVs have limitations as drugs—modest and variable target selectivity, finite intrinsic cargo, limited loading capacity, endosomal sequestration, and batch heterogeneity—that have motivated extensive engineering (Table 3) [56,58]. Cargo can be loaded endogenously by engineering producer cells to overexpress therapeutic proteins or RNAs, optionally fused to EV-sorting domains or targeted by EXOmotif-bearing sequences that exploit native sorting machinery [22], or exogenously after isolation by electroporation, sonication, extrusion, freeze–thaw cycling, saponin permeabilization, or passive incubation for compatible small molecules. Each exogenous method trades loading efficiency against membrane integrity and product purity: electroporation can load small RNAs but may aggregate cargo or vesicles, whereas passive incubation is gentler but often yields lower loading [56,58,59,60]. As an example of small-molecule loading into mammalian EVs, curcumin encapsulated in exosomes derived from EL-4 mouse lymphoma cells showed increased stability, systemic concentration, and anti-inflammatory activity relative to free curcumin [61].

EV surfaces can be decorated with targeting ligands, antibodies, nanobodies, aptamers, or homing peptides through genetic fusion to abundant membrane proteins (Lamp2b, tetraspanins, platelet-derived growth factor receptor transmembrane domains, or glycosylphosphatidylinositol anchors) or through post-isolation chemical conjugation, including copper-free click chemistry and lipid-anchor insertion [8,56,58,60]. Hybrid EV-liposome approaches attempt to combine biological membrane identity with the loading and formulation control of synthetic carriers. The practical constraints are common to these strategies: preserving vesicle integrity and identity, achieving reproducible ligand density and cargo copy number, improving productive cytosolic delivery, and demonstrating that the engineered feature—rather than a co-purified contaminant or process-related impurity—accounts for activity.

EVs should therefore be positioned alongside, rather than categorically above, established viral and synthetic delivery systems (Table 4). Compared with lipid nanoparticles (LNPs), EVs offer a biologically derived membrane containing native proteins and lipids that can support compatibility with diverse cargos and may provide useful cell-interaction cues, but LNPs generally offer more controllable nucleic-acid loading, composition, scalable manufacturing, and a more mature chemistry-manufacturing-control framework; both platforms can exhibit off-target organ uptake and incomplete endosomal escape [59,62]. Adeno-associated viruses (AAVs) can achieve highly efficient and durable transgene expression, but are constrained by payload size, anti-capsid immunity, and re-dosing challenges, whereas EVs can carry different molecular classes without viral genomes but often show lower and more variable functional delivery efficiency [62,63]. Polymeric nanoparticles and virus-like particles (VLPs) provide additional tunability or ordered protein-shell assembly, respectively, while introducing their own toxicity, immunogenicity, cargo, and manufacturing trade-offs [62]. The strongest case for EVs is therefore indication-specific: their biological membrane identity or engineerable cell interactions must provide value that justifies more difficult product characterization and batch control.

### 6.2. EV-Based Drug Delivery in Cancer

These strategies have produced several proofs of concept. Engineered exosomes carrying short interfering or short hairpin RNA against oncogenic KRAS (“iExosomes”) suppressed pancreatic tumor growth and improved survival in multiple mouse models, including genetically engineered models of pancreatic ductal adenocarcinoma, outperforming liposomes carrying the same RNA by exploiting EV-intrinsic tropism, CD47-mediated protection from phagocytic clearance, and macropinocytotic uptake by KRAS-mutant tumor cells [64]. This work has since advanced to first-in-human evaluation.

Earlier, surface display of the neuron-targeting rabies-virus glycoprotein (RVG) peptide fused to Lamp2b enabled systemic exosomal delivery of small interfering RNA across the blood–brain barrier, producing specific knockdown of BACE1 in mouse brain without measurable immune activation and providing a template for ligand-directed EV therapeutics [65]. Chemotherapeutic payloads have been delivered in the same manner: exosomes engineered to display the iRGD tumor-homing peptide and loaded with doxorubicin by electroporation achieved targeted accumulation and tumor growth inhibition with reduced cardiotoxicity relative to free drug [66]. EVs from a range of engineered sources are being developed for targeted chemotherapy, gene therapy, gene editing, photodynamic therapy, and immunotherapy [58,67].

### 6.3. EV-Based Immunotherapy

EVs have also been developed as immunotherapeutic agents. Dendritic-cell–derived exosomes bearing peptide–MHC class I and class II complexes, costimulatory molecules, and adhesion proteins can prime T-cell responses and eradicated established murine tumors in an early cell-free vaccine study [68]; subsequent work showed that these vesicles frequently act indirectly, by transferring antigen–MHC complexes to recipient dendritic cells that then activate naïve CD4+ T cells, which clarified their mechanism and informed vaccine design [69]. Dendritic-cell exosome (“dexosome”) vaccines have been tested clinically: a first-in-human phase I trial in metastatic melanoma demonstrated feasibility and an absence of significant toxicity [70], and a phase II trial of interferon-γ–matured dexosomes as maintenance immunotherapy after first-line chemotherapy in non-small cell lung cancer confirmed safety and showed natural killer cell activation, although it did not meet its primary progression-free survival endpoint [71]. These trials indicate that allogeneic and autologous EV products are clinically tolerable, and that the main limitation is potency rather than safety.

More recently, the recognition that exosomal PD-L1 drives immune evasion has opened complementary strategies: genetic or pharmacological suppression of exosomal PD-L1 secretion, extracorporeal removal of circulating tumor EVs by affinity hemofiltration, and engineering of EVs to display checkpoint-blocking or agonistic immune ligands, each potentially synergizing with antibody checkpoint inhibitors [45,50]. EV-based delivery of cytokines, stimulator of interferon genes (STING) agonists, and tumor antigens is likewise under active development.

### 6.4. Engineering EVs for Regeneration

In regenerative medicine, the same methods are used to enhance repair: loading EVs with pro-angiogenic or anti-inflammatory miRNAs and proteins, overexpressing growth factors or transcription factors in producer cells, or displaying tissue-homing peptides such as bone-, cartilage-, or ischemic-myocardium–targeting sequences, increases potency and target specificity while reducing the dose required. Combining surface targeting with the sustained-release scaffolds discussed in Section 3.4 addresses both of the principal pharmacological weaknesses of native EVs. Functionalized EVs are now pursued in both cancer and regenerative therapy using a common engineering platform [35,56].

### 6.5. Plant-Derived Exosome-like Nanovesicles

A more recent direction is the use of plant-derived exosome-like nanovesicles (PELNVs). Isolated from fruits, vegetables, and herbs, these nanoscale particles contain lipids, RNAs, proteins, and bioactive plant metabolites and have shown anti-inflammatory, antioxidant, tissue-protective, and antitumor activities in preclinical studies [72]. PELNVs are relevant to the cancer-regeneration interface because a single source class can modulate inflammatory and mucosal-repair pathways while also influencing tumor biology or serving as a carrier for anticancer drugs. They also provide a useful manufacturing contrast to mammalian EVs: abundant plant biomass and oral formulations may support low-cost scale-up, but plant sourcing introduces distinct questions of nomenclature, biogenesis, cultivar and seasonal variability, potency definition, and mammalian immunological or toxicological assessment. Avoiding mammalian producer cells removes some source-related concerns but does not eliminate the need for rigorous safety and product characterization.

Preclinical evidence illustrates this interface. Lipids extracted from grapefruit-derived nanoparticles were reassembled into carriers that delivered chemotherapeutic agents, small interfering RNA, DNA expression vectors, and proteins to several tissues with low reported toxicity and could be redirected using inflammation-homing ligands [73]. Edible ginger-derived nanoparticles administered orally were preferentially taken up by intestinal epithelial cells and macrophages, reduced acute colitis, enhanced mucosal healing, and attenuated colitis-associated cancer in mice [74]. This model is especially relevant to the theme of this review because it links tissue repair and control of inflammation-driven tumorigenesis within the same disease continuum. Together, these studies suggest that PELNVs may bridge regenerative and oncological applications, although standardized nomenclature, source-dependent compositional variability, reproducible loading and potency, and rigorous evidence that plant RNAs or other constituents act on defined mammalian targets remain open issues [72].

### 6.6. Manufacturing, Scale-Up, and Quality Control

Translation of these strategies requires manufacturing capacity beyond typical academic workflows. Ultracentrifugation-based isolation from flask-scale culture is neither readily scalable nor ideal for GMP production; clinical manufacturing increasingly uses well-characterized or banked producer cells in defined serum-free or EV-depleted media, scalable hollow-fiber or stirred-tank bioreactors, and downstream tangential-flow filtration and chromatographic polishing [42,60,75]. Each unit operation must be controlled for vesicle recovery, impurity clearance, and preservation of biological activity, and process scale-up requires comparability testing rather than assuming that EVs produced at different scales are equivalent. Stable producer lines and controlled bioreactor conditions can improve yield and consistency, but engineering the producer cell also introduces requirements for genetic stability, substrate qualification, and monitoring of process-dependent changes in EV composition.

Quality control presents a further challenge. A release specification for an EV product should define identity, purity (including residual host–cell DNA, proteins, lipoproteins, endotoxin, and process reagents), quantity using validated particle or mass metrics, and potency in a bioassay that reflects the intended mechanism rather than relying on total protein alone [23,42,75]. Engineered products require additional critical quality attributes such as cargo amount or copy number, ligand density, membrane integrity, residual conjugation reagents, and evidence that the engineered modification remains stable during storage. Freeze–thaw cycling, concentration, and lyophilization can alter vesicle size distribution or activity, so storage formulations and cryoprotectants must be qualified for each product. Regulatory classification can also vary with source, manipulation, and claimed mechanism; regulators have cautioned against unapproved commercial ‘exosome’ products lacking such evidence [32,58,67].

Recent translational analyses emphasize that engineering can address key bottlenecks but simultaneously increases chemistry-manufacturing-control complexity [60,75]. Defined cargo-sorting systems and producer-cell engineering can improve loading and potency; surface ligands can improve target engagement; biomaterial or hybrid formulations can alter residence time and delivery; and formulation engineering can improve storage stability. Yet every added feature creates new variables that must be controlled. Post-isolation conjugation requires specifications for ligand density and reagent clearance, producer-cell genetic engineering moves complexity upstream into cell-bank and genetic-stability control, and EV-liposome or EV-LNP hybrids may improve loading while complicating product identity and potency assays. Engineering should therefore be selected to solve a defined translational failure mode—such as insufficient loading, targeting, residence time, or stability—rather than added by default. This design-for-translation approach links engineering choices directly to scalability, product consistency, potency, safety, storage, and regulatory characterization [60,75].

## 7. Conclusions

Extracellular vesicles occupy a productive conceptual interface between regenerative medicine and cancer biology. The comparison shows that EV activity is a systems property rather than a fixed attribute of the vesicle: producer-cell state, cargo, membrane and surface composition, biomolecular corona, dose and route, biodistribution, and recipient-cell context jointly determine outcome. Accordingly, angiogenesis, immune modulation, matrix remodeling, and pro-survival signaling can represent therapeutic potency in injured tissue yet become tumor-supportive liabilities in a different context. This bidirectional framework also enables practical exchange between the fields, from oncology-derived targeting and molecular analytics to regenerative approaches for local delivery, potency testing, and manufacturing.

The remaining barriers are biological as well as technical and regulatory. Fundamental questions persist regarding EV heterogeneity, the fraction of internalized cargo that is productively delivered, cell-specific uptake, quantitative dose–response relationships, the stability of engineered phenotypes, and context-dependent switching between regenerative and tumor-promoting effects. In parallel, clinical translation requires MISEV-aligned product definition, scalable and reproducible GMP manufacturing, mechanism-linked potency assays, quantitative biodistribution, qualified storage conditions, and product-specific safety frameworks. Progress will depend on integrating these biological and chemistry-manufacturing-control questions rather than treating EVs as a uniformly advantageous carrier class.

## Figures and Tables

**Table 1:** Principal biogenesis-based classes and operational size terminology for extracellular vesicles. Per MISEV2023, ‘small EV’ and ‘large EV’ are physical/operational descriptors and are not synonymous with exosomes or microvesicles. Listed markers are EV-associated and do not, individually or collectively, establish exosomal or other biogenetic origin without complementary evidence.

EV Category	Biogenesis/Operational Definition	Typical Size	Representative EV-Associated Features
Exosomes	Endosomal origin: intraluminal vesicles form in multivesicular bodies and are released after fusion with the plasma membrane (ESCRT-dependent and/or independent). Biogenetic assignment requires evidence beyond size or common EV markers.	Commonly ~30–150 nm; size alone is not diagnostic	CD9, CD63, CD81, TSG101, ALIX, syntenin (EV-associated; not proof of exosomal origin)
Microvesicles/ectosomes	Direct outward budding from the plasma membrane; assignment requires evidence of plasma-membrane budding rather than size alone.	Often ~100–1000 nm; overlaps with other EV classes	Integrins, selectins, ARF6, flotillin (context-dependent, not uniquely diagnostic)
Apoptotic bodies	Membrane blebbing and fragmentation during apoptosis	Often ~1–5 µm; size distributions can overlap	Phosphatidylserine, histones, organelle/DNA remnants

**Table 2:** Representative methods for the isolation and characterization of extracellular vesicles.

Method	Principle	Relative Purity	Yield/Throughput	Key Notes
Differential ultracentrifugation	Sequential sedimentation by centrifugal force	Moderate	High yield, low throughput	Common reference method; co-isolates protein aggregates
Density-gradient ultracentrifugation	Flotation to equilibrium density	High	Low yield, low throughput	Improved purity; laborious
Size-exclusion chromatography	Separation by hydrodynamic size	High	Moderate	Preserves EV integrity; dilute eluate
Polymer precipitation	Volume exclusion (e.g., PEG)	Low	High yield, high throughput	Fast and scalable; co-precipitates contaminants
Immunoaffinity capture	Antibody binding to surface markers	Very high (subset)	Low	Subtype-specific; capture bias and cost
Microfluidic platforms	On-chip capture/sorting (passive or active)	High	Low volume, automatable	Rapid and reproducible; emerging for clinical use

**Table 3:** Representative engineered and source-based extracellular vesicle therapeutic strategies.

Strategy	Approach	Representative Example	Application
Endogenous cargo loading	Producer-cell engineering to package RNA/protein	iExosomes carrying anti-KRAS siRNA	Pancreatic cancer
Surface ligand targeting	Display of homing peptides or antibodies	RVG-peptide exosomes for siRNA	CNS-targeted gene therapy
EV-based vaccine	Antigen-presenting, DC-derived exosomes	Dendritic-cell exosome vaccine	Anti-tumor immunity
Checkpoint modulation	Depletion or blockade of exosomal PD-L1	Suppression of exosomal PD-L1	Immunotherapy sensitization
MSC-EV regenerative therapy	Native or preconditioned MSC EVs	Wound and cartilage repair	Regenerative medicine
Plant-derived nanovesicles	Botanical exosome-like vesicles	Fruit/vegetable/herb ELNs	Sustainable delivery and anti-inflammation

**Table 4:** Comparison of extracellular vesicles with representative synthetic and viral delivery platforms. The relative strengths and limitations are indication- and formulation-dependent [59,62,63].

Platform	Cargo/Loading	Endosomal Escape & Functional Delivery	Targeting/Biodistribution	Scale, Consistency & Characterization
EVs	Proteins, RNAs, small molecules; endogenous or post-isolation loading; loading can be variable	Endosomal sequestration is common; productive cytosolic delivery can be limiting	Native surface cues can be useful but tropism is context-dependent; IV dosing often favors clearance organs	Biological identity is complex; scalable production and batch consistency remain major challenges
LNPs	High and controllable nucleic-acid encapsulation; formulation is compositionally defined	Ionizable lipids can promote escape, but only a fraction of internalized cargo reaches the cytosol	Strong formulation- and route-dependent organ bias; targeting ligands can be added	Highly scalable mixing/formulation; comparatively mature CMC and analytical framework
AAVs	DNA payload with limited packaging capacity; highly efficient gene transfer	Viral trafficking supports efficient nuclear delivery in permissive cells	Capsid tropism can be strong but may be broad or tissue-limited; receptor dependence matters	Established vector manufacturing, but capsid immunity, re-dosing, payload size, and vector QC are constraints
Polymers	Chemistry can be tuned for diverse cargos and release profiles	Escape can be engineered but varies widely with polymer chemistry and toxicity	Ligands and physicochemical properties are readily tunable	Synthetic scale-up can be straightforward, but polydispersity, toxicity, degradation, and formulation reproducibility require control
VLPs	Ordered protein shells can package or display defined cargos/antigens	Cell entry can be efficient; intracellular routing depends on VLP design	Protein-shell engineering enables receptor targeting but can add immunogenicity	Recombinant manufacturing is established for some systems; payload, assembly, immunity, and structural characterization are key constraints

## Data Availability

No new data were created or analyzed in this study. Data sharing is not applicable to this article.
